# The Prevalence and Clinical Significance of Anaerobic Bacteria in Major Liver Resection

**DOI:** 10.3390/antibiotics10020139

**Published:** 2021-01-31

**Authors:** Jens Strohäker, Sophia Bareiß, Silvio Nadalin, Alfred Königsrainer, Ruth Ladurner, Anke Meier

**Affiliations:** Department of General, Visceral and Transplantation Surgery, University Hospital of Tuebingen, 72076 Tuebingen, Germany; Sophia.Bareiss@med.uni-tuebingen.de (S.B.); Silvio.Nadalin@med.uni-tuebingen.de (S.N.); Alfred.Koenigsrainer@med.uni-tuebingen.de (A.K.); Ruth.Ladurner@med.uni-tuebingen.de (R.L.); Anke.Meier@med.uni-tuebingen.de (A.M.)

**Keywords:** anaerobic infection, liver resection, cholangitis, biliary tract infection

## Abstract

(1) Background: Anaerobic infections in hepatobiliary surgery have rarely been addressed. Whereas infectious complications during the perioperative phase of liver resections are common, there are very limited data on the prevalence and clinical role of anaerobes in this context. Given the risk of contaminated bile in liver resections, the goal of our study was to investigate the prevalence and outcome of anaerobic infections in major hepatectomies. (2) Methods: We retrospectively analyzed the charts of 245 consecutive major hepatectomies that were performed at the department of General, Visceral, and Transplantation Surgery of the University Hospital of Tuebingen between July 2017 and August 2020. All microbiological cultures were screened for the prevalence of anaerobic bacteria and the patients’ clinical characteristics and outcomes were evaluated. (3) Results: Of the 245 patients, 13 patients suffered from anaerobic infections. Seven had positive cultures from the biliary tract during the primary procedure, while six had positive culture results from samples obtained during the management of complications. Risk factors for anaerobic infections were preoperative biliary stenting (*p* = 0.002) and bile leaks (*p* = 0.009). All of these infections had to be treated by intervention and adjunct antibiotic treatment with broad spectrum antibiotics. (4) Conclusions: Anaerobic infections are rare in liver resections. Certain risk factors trigger the antibiotic coverage of anaerobes.

## 1. Introduction

Liver resections are widely available procedures to cure benign and malignant diseases of the liver. After partial hepatectomy bile leaks and surgical site infections, complications are feared, since they drastically increase morbidity and mortality [1,2,3]. Liver resections are considered clean-contaminated procedures given the risk of preexisting bacterial and fungal colonization of the biliary system. The most common bacteria cultured from the bile duct and postoperative infectious complications are of gastrointestinal origin [4,5,6].

Anaerobic liver infections, however, are rare in hepatobiliary surgery. The most commonly described presentation of anaerobes in the liver is in the form of liver abscesses. These abscesses are frequently caused by acute or chronic abdominal inflammation or infection [7,8]. Recently, an emerging number of abscesses have been of iatrogenic origin, caused by percutaneous ablation of primary and secondary liver malignancies. Abscesses after ablation appear to develop in up to 2% of patients [9,10]. Anaerobic bacteria are common pathogens in the intestines. The liver and native biliary tract are generally uncommon habitats for anaerobic bacteria. However, they may be cultured from the biliary tract in up to 20% of patients in the presence of a biliary tract occlusion or stent [11,12]. Aside from biliary stents, bilioenteric anastomoses are considered a risk factor for anaerobic colonization and infection of the biliary tree (and liver). Therefore, the 2018 Tokyo Guidelines (TG2018) recommend covering anaerobic bacteria when treating cholangitis in the presence of bilioenteric anastomosis [13].

The department of General, Visceral, and Transplantation Surgery of the Tuebingen University Hospital is a Tertiary Care Academic Teaching facility that performs an average of ~200 liver resections as well as ~50 liver transplantations per year. Our center is specialized on complex liver resections with a clinical and scientific focus on biliary malignancies. [14,15]. A major hepatectomy/liver resection is defined as the removal of ≥3 liver segments [16]. Due to biliary obstruction of perihilar malignancies, patients often present with jaundice or have undergone preoperative stenting and are thus at risk of biliary tract infections [12]. Infectious complications after surgery are still a major risk factor for morbidity and mortality after liver resection. Surgical site and organ-space infections present in up to 20% of major hepatectomies [1,17]. The role of anaerobic bacteria is yet to be assessed. To our knowledge, there are neither sufficient data on the presence of anaerobes in intraoperative microbiological specimen from liver resections nor from surgical site infection (SSI) after hepatectomy. Even less is known about the presence of anaerobic bacteria’s antibiotic resistance to commonly used antibiotics after liver surgery.

The goal of this study was to evaluate the prevalence and clinical role of anaerobic bacteria in patients undergoing major liver resection for benign and malignant disease.

## 2. Results

### 2.1. Clinical Characteristics

We analyzed the charts of all consecutive patients that had undergone major liver resection at the department of General, Visceral, and Transplantation Surgery of the Tuebingen University Hospital from June 2017 to August 2020. We included all adult (age ≥ 16 years) consecutive patients that underwent laparotomy and had at least three liver segments removed. Pediatric patients, as well as patients that had minor liver resections, were excluded. Based on the preoperative diagnosis and intraoperative findings, material was sent for microbiological testing at the attending surgeon’s discretion. 

During the study period, 245 patients met the inclusion criteria. Of these 245 patients, 76 had intraoperative material sent for microbiological testing, of which 49 showed growth of pathogenic bacteria. Furthermore, 102 of the 245 patients had culture-proven microbiological growth during the first 30 postoperative days (this includes the 49 that had growth on the intraoperative cultures). From these 102 patients, we were able to identify 13 patients that had anaerobic infections. Of these 13 patients, 7 patients had positive anaerobic cultures from specimens that were collected during under the primary surgical procedure. The remaining six patients had positive anaerobic cultures during revision (*n* = 5) procedures or from specimen collected from drains (*n* = 1). The median age was 60 years (Standard Deviation (SD) ±15, range 34–80). Five patients were male (38%), and eight were female (62%). Most patients underwent liver resection for malignancy (*n* = 9), while the other patients were treated for helminthic disease (*n* = 2), suspected cholangiocarcinoma (*n* = 1), and recurrent cholangitis after cholecystectomy (*n* = 1). Of the 13 patients, 9 patients received prolonged perioperative antibiotics for suspected contaminated biliary tract (*n* = 3) or risk of post-hepatectomy liver failure (PHLF) (*n* = 6). These patients had anaerobic coverage with metronidazole (*n* = 6) or piperacillin/tazobactam (*n* = 2) or meropenem (*n* = 1). For details, see Table 1.

### 2.2. Clinical Comparison of Anaerobic Infections

We compared the patient group that suffered from anaerobic infections to the control group, which consisted of the remaining 232 patients. Gender distribution (38% male vs. 56% male; *p* 0.204 Χ^2^) was similar in both groups. The same was true for age (60 years standard deviation (SD) ± 15 vs. 64 years SD ± 13; *p* 0.373 MWU) and body mass index (BMI) (25.2 kg/m^2^ SD ± 4.7 vs. 24.6 kg/m^2^ SD ± 4.6; *p* 0.415 MWU). There were similar rates of malignant (69% vs. 88%; *p* 0.052 Χ^2^) and infectious (8% vs. 15 %; *p* 0.429 Χ^2^) underlying disease. In the anaerobic group, there were significantly higher rates of preoperative stents (54% vs. 27%; *p* 0.000 Χ^2^), bile duct resections and reconstructions (69% vs. 32%; *p* 0.006 Χ^2^), and bile leaks (54% vs. 18%; *p* 0.001 Χ^2^). Mean operating time was longer (*p* 0.035 MWU) in the anaerobic group (likely due to the higher rate of bile duct reconstructions), as well as the length of stay (*p* 0.002 MWU). The attending surgeon decided whether to activate postoperative preventive measures to preemptively treat post-hepatectomy liver failure in 78% of patients of the anaerobic group and 63% of the control group (*p* 0.390 Χ^2^). For details, see Table 2.

### 2.3. Microbiological Results

**Figure 1:**
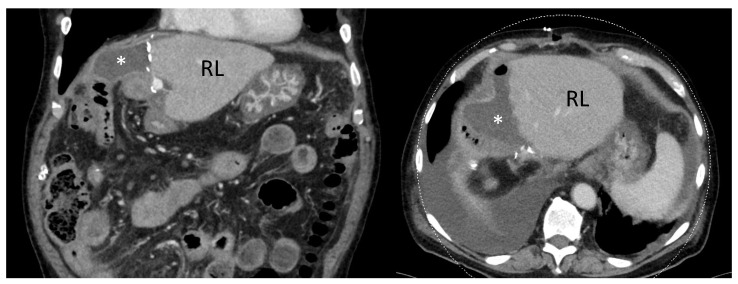
The CT scans of a patient that suffered from post-hepatectomy bilioma (white asterisk) originating from the remnant liver (RL). The left half of the image shows frontal/coronal plain, and the right half of the image shows the transverse plain.

Of the 245 patients in this study, 102 had positive microbiological cultures throughout their hospital stay and 13 patients grew anaerobic bacteria. Of these 13 patients, 7 patients had cultures from intraoperatively collected bile specimen. The remaining 6 patients had positive cultures from Bilioma (*n* = 2), perihepatic abscess (*n* = 1), subcutaneous abscess (*n* = 2), and enteric anastomotic leak (*n* = 1). Overall, there were 14 different strains of anaerobic bacteria cultured from these patients, and 1 patient had anaerobic bacteri, that we were unable to identify. *Prevotella* species was the most commonly isolated anaerobic species (*n* = 5; 33%), followed by *Bacteroides* strains (*n* = 3; 20%) and *Finegoldia magna* (*n* = 3; 20%). The latter was only cultured from specimen collected from “complications,” i.e., one bilioma (Figure 1), one perihepatic, and one subcutaneous abscess. Except for the perihepatic abscess caused by *Finegoldia magna*, all specimen that grew anaerobes showed polymicrobial growth of anaerobic and (multiple) aerobic bacteria. For details, see Table 3.

### 2.4. Risk Factor Analysis for Anaerobic Infection

Anaerobic infections were present in 13 out of 245 patients (5%). We performed a stepwise backward binary logistic regression to find risk factors for anaerobic infections in liver resection. As potential predictors, we chose age, BMI, diabetes mellitus 2, preoperative biliary stent, resection and reconstruction of the bile ducts, postoperative bile leak, extent of surgical procedure, American Society of Anesthesiologist (ASA) score, Model of End-Stage Liver Disease (MELD) score, activation of the in-house post-hepatectomy liver failure protocol, malignant disease, and infectious reason for surgery. In the final model, preoperative biliary stent and postoperative bile leak were identified as predictors for anaerobic infections, Χ^2^ (3) = 17.017, *p* 0.001; Cox & Snell 0.072; R^2^ Nagelkerke R^2^ 0.212. Preoperative stent had an Odds Ratio of 7.790 (CI 95% 2.151–28.221; *p* 0.002), while bile leak had an Odds Ratio of 5.426 (CI 95% 1.535–19.182; *p* 0.009).

### 2.5. Treatment of Anaerobic Infections

Of the 13 patients, 7 patients had positive biliary cultures from intraoperative specimens gathered from the bile duct. Six of these patients had a stent, and the remaining were suffering from repeated cholangitis after complicative cholecystectomy and injury of the right hepatic artery. All of these patients had preemptive perioperative coverage of anaerobic bacteria with metronidazole or piperacillin/tazobactam. All of these patients had their bile ducts and or infected segments of the liver resected. None of these patients had recurrent anaerobic infection during their stay.

In the other six patients, the most common sites of origin of anaerobic bacteria were the skin and gastrointestinal tract in 50% and 50% of patients, respectively. In order to resolve the infectious complications, surgical source control was achieved by evacuation of the infected tissues via incision, resection and drainage. None of these complications occurred. Overall, the outcomes were favorable. The overall mortality in the cohort was 6%. The mortality within the group of patients that suffered from anaerobic infections was 15%. However, none of the deaths was attributed to septicemia from anaerobic bacteria.

## 3. Discussion

The role of anaerobic bacteria in infections of the liver is unclear. The most commonly described pathologies are (septic) liver abscesses originating from the biliary tract, gastrointestinal tract, or endocarditis [7,8]. In 1996, Huang et al. reported that up to 25% of liver abscesses grew anaerobic bacteria [8]. The widespread use of percutaneous ablation of liver tumors has also led to an increase in liver abscesses caused iatrogenically [9,10], especially if the patient has undergone previous bilioenteric anastomosis [18,19]. 

Data on the prevalence of anaerobic bacteria and their role in complications after liver resections are scarce. Surgical site and organ space infections after liver resections pose an increased risk to the patient and significantly prolong hospital stay [20]. We hereby present the first study to focus on the prevalence of anaerobes in microbiological specimen collected during major liver resection and complications following these procedures. We present a series of 245 consecutive patients who underwent major liver resection at our tertiary hepatobiliary center. We were able to identify seven patients with anaerobic bacteria in the bile duct, as well as six patients that developed complications and subsequent anaerobic infections. Unsurprisingly, the most common site of origin of these anaerobes was in the gastrointestinal tract. Therefore, a breach of the natural barrier at the duodenal papilla appears to be a risk factor for anaerobic colonization of the biliary tract, similar to fungal colonization of the bile duct [12]. The number of anaerobic infections appears to increase the longer a stent is in place [12]. Even though a recent meta-analysis suggests that there are less infections with percutaneous drainage of the biliary tract compared to endoscopic drainage, the rates of the bacterial colonization were high in both groups [5,21]. Biliary drainage prior to surgery leads to an increased risk of postoperative infection [22,23]. In a cohort of 475 patients who had undergone preoperative biliary drainage, Sugawara et al. reported that nearly 75% of patients had contaminated bile. Of these patients, 28.6% developed postoperative infectious complications. Bacteriobilia is an independent risk factor of postoperative infectious complications. However, compared to the endoscopic internal drainage, Sugawara et al. did not report a single positive anaerobic culture [5,12].

Perioperative anti-infective treatment in liver surgery is usually done as a single dose prior to incision, with repeated doses based on the length of the procedure and excretion kinetics of the substance used [24]. There are little data that support prolonged anti-infective treatment unless patients are operated in the setting of an infection [24]. Our in-house protocol recommends perioperative antibiotics for a minimum of 3 days in patients who had undergone preoperative stenting or recurrent cholangitis or are at an increased risk of post-hepatectomy liver failure. We frequently cultured bacteria from the bile that were not susceptible to first- and second-generation cephalosporins and thus elected to use third-generation cephalosporins for liver and bile duct surgery. All patients undergoing biliary tract surgery receive perioperative third-generation cephalosporins with metronidazole (and fluconazole in case of a stent). External drainage is very uncommon in our region. Therefore, our perioperative regimen is based on the known local resistance patterns. Whereas there are sufficient data to not recommend prolonged antibiotics in elective and “sterile” liver surgery, there are less data on what to do in the setting of a contaminated bile duct. Sudo et al. found an increased rate of antibiotic resistance in patients that had undergone biliary drainage compared to those who had not. This may in part be due to preoperative antibiotic treatment and stent placement in the setting of cholangitis. Whereas there is a risk of bias, we support their suggestion to take into account microbiological culture results from specimens gathered from external bile drainage whenever available [25]. A Japanese randomized controlled trial confirmed that culture-based targeted therapy leads to less surgical site infections [26]. The most frequently reported bacterial species are E. coli and Enterococcus [4,5,23]. There were no reports on anaerobic growth in these trials.

We hypothesized that infected biliomas are frequently colonized by anaerobic bacteria. In our study, we were able to culture anaerobic bacteria from two biliomas with polymicrobial infection. The bacterial spectrum of biliomas after liver surgery is considered to be similar to that of cholangitis. A single retrospective analysis of 32 biliomas (most of them after liver surgery) reported the microbiological spectrum of these patients. The authors isolated 121 different bacterial strains from 32 biliomas. Only four of these were anaerobes (all *Clostridium perfringens*) [4]. A higher rate of up to 10% of biliomas and liver abscesses infected with anaerobes was reported in liver transplant recipients [27,28]. To our knowledge, there is no larger study available that has focused on microbiological data on bilioma after liver resection. Thus, the incidence of anaerobic bacteria in this population is yet to be assessed.

Multicentric randomized trials need to be conducted to solve the question of the true prevalence of anaerobic infections in liver resections. Aside from bile and tissue cultures, new but not yet widely available diagnostic tools need to be introduced. A recent study by Dyrhovden et al. from Norway examined next generation sequencing from biliary samples in cholecystitis [11]. They reported up to 23% of anaerobic bacteria in bile samples from gallbladder aspirates and lower numbers from cultures. This suggests that we need to change the way we think about microbiological sampling during surgical procedures. There are no recent studies that have compared tissue and aspirate samples to swabs from infected tissues in abdominal surgery. Most studies comparing different sampling techniques have focused on chronic wounds [29] and infected musculoskeletal tissues [30]. Swabs from infected tissues are still a common specimen sampling in ORs worldwide. Clinicians and microbiologists will need to focus more on the proper collection and work up of samples from abdominal infections. Whereas the bile itself may be inhibitory to the growth of bacteria, we frequently had polymicrobial cultures [31]. The delayed growth of anaerobes and fungi furthermore may lead to therapeutic insecurity, especially when cultures grow bacteria days after surgery that are not covered by empiric antibiotics. Next-generation sequencing (NGS) potentially offers earlier results than cultures. In the near future, NGS may help in the decision process of when to stop empiric antibiotics early, thus preventing resistance formation and limiting unnecessary toxicity and costs. 

Overall, merely 5% of our patients developed anaerobic infections. Assuming Dyrhovden’s results could be transferred to major hepatectomies, we are at risk of significant underdiagnosis [11]. In our patient cohort, preoperative biliary stents and postoperative bile leaks were predictors of anaerobic infection. This is similar to our department’s finding that anaerobic bacteria were more frequently isolated in cholecystitis patients who had undergone preoperative endoscopic retrograde cholangiopancreaticography [32]. The 2018 Tokyo Guidelines recommend covering anaerobes in the setting of cholangitis in patients that have biliary obstruction or bilioenteric anastomosis [13]. We therefore continue to empirically treat anaerobes in all hepatectomies involving biliary tract resections, preoperative stenting, and postoperative bilioma and bile leaks. Further prospective data are needed to support this approach.

The most commonly used antibiotics to treat anaerobes in abdominal infections are metronidazole, piperacillin/tazobactam, and carbapenems [13]. According to a 2020 review by Thabit, all three options have excellent penetration into the peritoneum as well as the biliary tract. However, since there are limited data on the biliary penetration of ertapenem and due to its intrinsic lack of activity against *pseudomonas*, meropenem or imipenem/cilastatin are likely to be preferred in infections of the liver [33]. Clindamycin, which is perceived to be a good candidate to treat anaerobes in the skin and “above the diaphragm,” has shown decreased activity against gastrointestinal aerobe and anaerobe pathogens [34] and has fallen out of the general surgeon’s favor for the increased risk of *clostridium difficile* infection [35] compared to other antibiotics.

### Strengths and Limitations

To our knowledge, this is the first study to focus solely on the prevalence and clinical presentation of anaerobic bacteria in major liver resection. We presented data from 245 cases of major liver resections from our tertiary hepatobiliary surgery unit. We were able to identify biliary stenting and bile leaks as predictors for anaerobic infections. However, given the overall low prevalence of anaerobes, caution must be taken when generalizing these results. Overall, we were slightly surprised to find only 5% of anaerobic infections over this 3-year period. Given the high rate of cholangiocarcinoma in our cohort and the consecutive increased rate of bile duct obstructions and bile leaks, we expected to find a higher number of positive anaerobic cultures. Due to the retrospective character of this study, we were unable to optimize specimen sampling and were also unable to perform next-generation sequencing to correlate these results to those of our cultures.

## 4. Materials and Methods

### 4.1. Data Acquisition

We retrospectively screened our hospital information system for all patients who underwent major liver resection at the department of General, Visceral, and Transplantation Surgery of the University Hospital of Tuebingen, Germany. All adult patients (age ≥ 16 years) who underwent resection of at least 3 liver segments as an individual procedure at our center between January July 2017 and August 2020 were included in the final analysis. The medical reports of these patients were screened for intra- and perioperative microbiological cultures. Cultures were sent at the surgeon’s discretion, most commonly when there were clinical signs of chronic cholangitis or biliary stenting or intraoperative findings of abscesses or perforation. The study was performed on a consecutive database and was approved by the local ethics committee under the reference number 419/2020/BO.

### 4.2. Clinical Definitions 

Major hepatectomy/liver resection is defined as the simultaneous resection of at least 3 segments of the liver. A right hepatectomy is the removal of Coinaud’s segments V-VIII, a left hepatectomy is the removal of Segments II-IV, a right Trisectionectomy is the removal of Segments IV-VIII, and a left Trisectionectomy is the removal of liver segments II-V and VIII according to the 2000 Brisbane terminology [16]. A bilioma is defined as an organized collection of bile, usually with association to the liver resection margin or to the bilioenteric anastomosis that is encapsulated and filled with bile.

### 4.3. Isolation and Identification of Strains

All samples were sent for both aerobic and anaerobic cultures. For the anaerobic cultures, we used a custom-built highly enriched and supplemented Sheep Blood Agar. Therefore, sheep blood (5% defibrinated, Source: Acila) was added to brain-heart infusion agar (Source: Oxoid) and then enriched with IsoVitalex enrichment (Source: BD) at 37 °C. The method has been recently described by our microbiology department [36]. The isolates were then identified using Matrix-Assisted Laser Desorption/Ionization-Time Of Flight (MALDI-TOF) mass spectrometry.

### 4.4. Statistics

Comparison between groups was carried out by the Chi-Square test (Χ^2^) or Fisher´s exact test (FET) for nominal variables and the Mann–Whitney U-test (MWU) for continuous variables, as appropriate. A probability of less than 0.05 was considered to be statistically significant. All p-values reported were the result of 2-sided testing. Where needed, Bonferroni correction was applied. Statistical analysis was carried out using IBM SPSS Statistics for Windows, Version 26.0 (IBM Corp., Armonk, NY, USA).

## 5. Conclusions

Anaerobic infections play an unknown rule in the setting of major hepatectomy. This study found an increased rate of anaerobic infection in patients that had undergone preoperative endoscopic biliary stenting. Furthermore, postoperative bile leaks appeared to be a predictor of anaerobic infections. Currently, there are little to no available trials that report the rate of anaerobic infections in hepatobiliary surgery, which may in part be due to the slow and difficult growth rate of anaerobes. Whereas the outcomes of the anaerobic infections in our cohort were favorable overall, all of our postoperative complications caused by anaerobic bacteria had to be treated interventionally and/or surgically in addition to the anti-infective medication. Further data is needed to understand the role of anaerobic bacteria in patients undergoing liver resections and to predict the presence and role of anaerobes in complications after hepatectomy. Newer diagnostic tools, such as next-generation sequencing, may aid in the early and precise identification of anaerobic strains.

## Figures and Tables

**Table 1:** The surgical procedures and outcomes, as well as the isolated anaerobic strains of the 13 patients that had positive anaerobic cultures. * Procedures according to Brisbane classification.

Surgical Details and Outcome								
Age, Gender	Diagnosis	Procedure	Location of Specimen	Anaerobe	Preoperative Stent	Complications	Length of Stay	Outcome
34 f	iCCA	Right Trisectionectomy * + Resection of Extrahepatic bile duct and bilioenteric anastomosis	Bile duct 1st Procedure	Prevotella buccae Prevotella melaninogenica	Yes		11d	alive
72 m	HCC	Right Trisectionectomy + Resection of Extrahepatic bile duct and bilioenteric anastomosis	Bile duct 1st Procedure	Anaerobe not otherwise specified	Yes	death from septic shock due to post-hepatectomy liver failure	29d	dead
44 w	Embryonal Sarcoma of the Liver	Right Trisectionectomy + Segmental Colectomy	Anastomotic leak Revision	Bacteroides vulgatus	No	death from multi-organ failure with bile leak and peritonitis & post-hepatectomy liver failure	35d	dead
38 m	Echinococcus alveolaris	Right Trisectionectomy + Resection of Extrahepatic bile duct and bilioenteric anastomosis	Bile duct 1st Procedure	Bifidobacterium animalis	Yes		28d	alive
71 m	phCCA	Left Hepatectomy + Resection of Extrahepatic bile duct and bilioenteric anastomosis	Bile duct 1st Procedure	Bacteroides thetaiotaomicron	Yes		13d	alive
78 f	phCCA	Left Hepatectomy + Resection of Extrahepatic bile duct and bilioenteric anastomosis	Bile duct 1st Procedure	Prevotella buccae	Yes		20d	alive
40 m	Echinococcus granulosus	Right Hepatectomy	Abscess perihepatic and subcutaneous Revision	Finegoldia magna	No	perihepatic abscess, wound dehiscence	29d	alive
80 m	Klatskin-mimicking Lesion	Right Trisectionectomy + Resection of Extrahepatic bile duct and bilioenteric anastomosis	Bilioma CT-Drain	Prevotella melaninogenica	Yes	infected bilioma	35d	alive
68 f	GBCA	Right Trisectionectomy + Resection of Extrahepatic bile duct and bilioenteric anastomosis	Bile duct 1st Procedure	Bilophila wadsworthia Veillonella parvula	Yes	revision laparotomy for postoperative bleeding	31d	alive
54 f	Recurrent Cholangitis	Right Hepatectomy + Resection of Extrahepatic bile duct and bilioenteric anastomosis	Bile duct 1st Procedure	Bacteroides fragilis	No		8d	alive
58 f	iCCA	Right Trisectionectomy + Resection of Extrahepatic bile duct and bilioenteric anastomosis	Deep subcutaneous abscess	Prevotella buccae	No	revision laparotomy for postoperative bleeding	25d	alive
60 f	NET-Metastasis	Right Trisectionectomy + Resection of Extrahepatic bile duct and bilioenteric anastomosis	Deep subcutaneous abscess	Finegoldia magna	No	revision laparotomy for postoperative ileus	36d	alive
60 f	CRLM	Left Hepatectomy	Bilioma Revision	Finegoldia magna	No	revision laparotomy for postoperative bile leak	21d	alive

**Table 2:** The comparison of the group of patients that grew anaerobic bacteria and the control group that did not.

Anaerobic Group vs. Control Group			
	Anaerobes (*n* = 13)	No Anaerobes (*n* = 232)	*p*
Gender m (Percentage)	5 (38%)	131 (56%)	0.204
Median age in years	60 (± 15)	64 (± 13)	0.373
Body mass index (BMI) in kg/m^2^	25.2 (± 4.7)	24.6 (± 4.6)	0.415
Diabetes	3 (23%)	35 (15%)	0.439
Diagnosis Malignant Recurrent infections	9 (69%) 1 (8%)	204 (88%) 35 (15%)	0.052 0.429
Preoperative biliary stent	7 (54%)	85 (27%)	0.000
Procedure Right Hepatectomy Right Trisectionectomy Left Hepatectomy Left Trisectionectomy Central Resection	2 (15%) 8 (62%) 3 (23%) 0 0	91 (39%) 75 (32%) 33 (14%) 25 (11%) 5 (2%)	0.114
Median operating time in minutes Median length of stay in days	292 (± 73) 28 (± 9)	247 (± 100) 13 (± 13)	0.035 0.002
Resection of extrahepatic bile duct	9 (69%)	75 (32%)	0.006
Bile leak	7 (54%)	41 (18%)	0.001
Elevated risk for PHLF	10 (78%)	147 (63%)	0.390

**Table 3:** The isolated bacterial strains.

Microorganisms Isolated		
	*n*	%
*Bacteroides fragilis* *Bacteroides thetaiotaomicron* *Bacteroides vulgatus* *Prevotella buccae* *Prevotella melaninogenica* *Finegoldia magna* *Bilophila wadsworthia* *Veillonella parvula* *Bifidobacterium animalis* *Anaerobe not otherwise specified*	1 1 1 3 2 3 1 1 1 1	6.7 6.7 6.7 20.0 13.3 20.0 6.7 6.7 6.7 6.7
Total	15	100

## Data Availability

The data presented in this study are available on request from the corresponding author. The data are not publicly available du the pseudonymized character of the data.

## Abbreviations

ASA	American Society of Anesthesiologist
CRLM	Colorectal Liver Metastasis
BMI	Body Mass Index
CI	Confidence Interval
FET	Fisher´s Exact Test
iCCA	intrahepatic Cholangiocarcinoma
GBCA	Gallbladder Carcinoma
HCC	Hepatocellular Carcinoma
MALDI-TOF	Matrix-Assisted Laser Desorption/Ionization-Time Of Flight
MELD	Model of End-Stage Liver Disease
MWU	Mann-Whitney U-Test
NET	Neuroendocrine Tumor
NGS	Next-Generation Sequencing
phCCA	perihilar Cholangiocarcinoma
PHLF	Post-Hepatectomy Liver Failure
SD	Standard Deviation
SSI	Surgical Site Infection
Χ^2^	Chi-Square test
